# Selective Mid-Wall Cardiac Dysfunction in Obesity: The Role of Muscle-to-Fat Balance

**DOI:** 10.3390/biomedicines13123083

**Published:** 2025-12-14

**Authors:** Karolina Angela Sieradzka Uchnár, Ingrid Schusterová, Štefan Tóth, Tibor Porubän, Mariana Dvorožňáková, Pavol Fülöp

**Affiliations:** 1 Department of Imaging Techniques, East Slovak Institute of Cardiovascular Diseases, Faculty of Medicine, Pavol Jozef Šafárik University in Košice, Ondavská 8, 040 01 Košice, Slovakia; sieradzka.ina@gmail.com (K.A.S.U.);; 2 Department of Gerontology and Geriatrics, Faculty of Medicine, Pavol Jozef Šafárik University in Košice, University Hospital of St. Michael, Murgašova 1, 040 86 Košice, Slovakia; 3 1st Department of Cardiology, East Slovak Institute of Cardiovascular Diseases, Faculty of Medicine, Pavol Jozef Šafárik University in Košice, Ondavská 8, 040 11 Košice, Slovakia; 4 2nd Department of Cardiology, East Slovak Institute of Cardiovascular Diseases, Faculty of Medicine, Pavol Jozef Šafárik University in Košice, Ondavská 8, 040 11 Košice, Slovakia

**Keywords:** skeletal muscle mass, obesity, cardiac strain, circumferential strain, muscle-to-fat ratio, sarcopenic obesity, young adults

## Abstract

**Objective:** This study aims to analyze relationships between body composition, biochemical parameters, and cardiac function in young adults to identify mechanisms of cardiac dysfunction in obesity. **Methods:** This is a cross-sectional study of 60 young adults (mean age 20.4 years) divided into healthy (*n* = 29) and overweight/obese (*n* = 31) groups. Body composition was assessed using bioelectrical impedance analysis. We calculated the SMM-to-Fat ratio (skeletal muscle mass %/body fat %) as a continuous composite metric. Cardiac function was evaluated using 3D speckle-tracking echocardiography, with a 3D global circumferential strain pre-specified as the primary endpoint. **Results:** The obese group showed unfavorable body composition with lower SMM% (38.0 ± 10.8 vs. 47.1 ± 5.6%), higher body fat% (28.3 ± 12.6 vs. 16.0 ± 8.3%), and lower SMM-to-Fat ratio (2.1 ± 2.3 vs. 4.8 ± 5.1; all *p* < 0.001). C-peptide was 75% higher (*p* < 0.001), indicating compensatory hyperinsulinemia. The primary endpoint showed impairment in the obese group (−19.8 ± 4.7 vs. −22.2 ± 2.9%; *p* = 0.023, *d* = 0.61), while longitudinal strain was preserved, indicating selective mid-wall dysfunction. The SMM-to-Fat ratio demonstrated a stronger association with circumferential strain (*r* = −0.467, *p* = 0.008) than SMM% alone (*r* = −0.414, *p* = 0.021) and remained an independent predictor in multivariable analysis (*β* = −0.88, *p* = 0.019), whereas SMM% did not achieve significance (*p* = 0.159). Comprehensive analysis revealed correlation reversal across all body composition parameters between groups, with minerals% and total body water% emerging as additional independent predictors. **Conclusions:** Young obese adults exhibit selective mid-wall cardiac dysfunction. The SMM-to-Fat ratio, representing muscle–adiposity balance, is superior to SMM% alone for predicting cardiac dysfunction. Our findings suggest that the relative balance, rather than absolute muscle mass, determines cardiac health in obesity, with implications for body composition assessment and intervention strategies.

## 1. Introduction

Global obesity rates have escalated to epidemic levels, with prevalence increasing approximately three-fold from around 12% in 1975 to beyond 38% in 2022 [1]. The World Health Organization forecasts that maintaining current trajectories will result in over 1 billion individuals worldwide living with obesity by 2030, constituting roughly 12% of the global population [2]. Conventional assessment strategies have emphasized body mass index (BMI) and adipose tissue accumulation as primary metrics, yet accumulating evidence increasingly emphasizes the pivotal contribution of skeletal muscle mass (SMM) to cardiometabolic health [3,4]. This paradigm shift acknowledges that somatic composition, specifically the proportional relationship between muscle and adipose compartments, may demonstrate greater importance than absolute body weight in determining cardiovascular (CV) health trajectories [5].

Sarcopenic obesity, characterized by concurrent excessive adiposity and diminished muscle mass, exemplifies a particularly adverse metabolic phenotype that compounds the pathophysiological risks inherent to both conditions [4,6]. Skeletal muscle constitutes the body’s predominant insulin-responsive tissue, mediating approximately 80% of insulin-dependent glucose clearance [7]. Beyond this metabolic function, muscle tissue operates as an endocrine organ through secretion of myokines that modulate systemic inflammatory responses, insulin sensitivity, and CV function [8,9]. Consequently, muscle mass deficiency demonstrates independent associations with insulin resistance, dyslipidemia, sustained low-grade systemic inflammation, and elevated CV risk [10,11].

Among young adults, a population in which lifelong health patterns and metabolic trajectories become established, the interrelationship between somatic composition and CV health assumes particular significance for early preventive interventions [12]. However, obesity-associated cardiac impairment in this demographic frequently remains in subclinical stages, escaping detection by conventional diagnostic approaches such as ejection fraction assessment [13]. Contemporary echocardiographic methodologies, notably two-dimensional and three-dimensional speckle-tracking strain imaging, facilitate identification of subtle myocardial contractile alterations that antecede clinically apparent cardiac dysfunction [14,15]. These sophisticated imaging platforms enable differentiation among longitudinal (subendocardial), circumferential (mid-wall), and radial deformation patterns, potentially elucidating the specific pathophysiological mechanisms and myocardial layers affected by metabolic perturbations [16].

Despite increasing awareness of sarcopenic obesity’s clinical relevance, limited investigations have systematically evaluated associations between SMM and layer-specific cardiac dysfunction in young adult populations employing comprehensive three-dimensional strain assessment. Clarifying these relationships could guide targeted therapeutic strategies to preserve both muscular and cardiac function preceding development of irreversible CV pathology.

Investigations utilizing computed tomography and magnetic resonance imaging have established that relative muscle mass—normalized to adipose mass—demonstrates more-robust associations with metabolic health parameters than absolute muscle mass measurements [15,16]. Studies in aging cohorts have documented that the proportional relationship between lean and adipose tissue predicts CV events with greater accuracy than either parameter evaluated independently [17]. Nevertheless, the specific associations between muscle–adipose equilibrium and cardiac strain phenotypes in young obese populations remain uninvestigated.

Therefore, the aims of our study were to (1) comprehensively analyze differences in body composition, biochemical parameters, and multi-dimensional cardiac function between healthy young adults and those with overweight/obesity; (2) identify specific correlations between SMM percentage and layer-specific myocardial strain patterns in both groups; and (3) determine whether SMM or SMM-to-Fat ratio independently predicts subclinical cardiac dysfunction after adjustment for relevant confounders.

## 2. Materials and Methods

### 2.1. Study Design and Participants

We conducted a cross-sectional observational study at the East Slovak Institute of Cardiovascular Diseases between January 2023 and December 2023. Participants were voluntarily recruited through university student health services and local community advertisements. A total of 78 individuals were screened for eligibility.

Inclusion criteria: (1) age 15–25 years; (2) BMI 18.5–24.9 kg/m^2^ for the control group or BMI ≥ 25 kg/m^2^ for the study group; (3) stable body weight (±2 kg) for at least 3 months; (4) ability to provide written informed consent.

Exclusion criteria: (1) known CV disease, including hypertension (systolic BP ≥ 140 mmHg or diastolic BP ≥ 90 mmHg), arrhythmias, or structural heart disease; (2) diabetes mellitus or fasting glucose ≥ 7.0 mmol/L; (3) chronic kidney or liver disease; (4) thyroid disorders; (5) pregnancy or lactation; (6) current use of medications affecting metabolism or cardiac function (including beta-blockers, angiotensin converting enzyme inhibitors, statins, or anti-diabetic medications); (7) professional athletes or individuals engaged in competitive sports (defined as membership in organized sports clubs with regular competition schedules, excluding recreational or school-level participation); (8) poor echocardiographic image quality precluding strain analysis.

All participants denied current tobacco smoking or use of nicotine-containing products. This homogeneity regarding smoking status eliminates smoking as a potential confounding variable in our analysis of the relationships between body composition and cardiac function.

Overall, 18 individuals were excluded: 8 due to inadequate echocardiographic windows for 3D strain analysis, 6 for medication use, 3 for thyroid disorders, and 1 for newly diagnosed diabetes. The final cohort comprised 60 young adults divided into two groups: control group (*n* = 29; mean age 21.0 ± 2.9 years) consisting of healthy individuals with BMI 18.5–24.9 kg/m^2^ and study group (*n* = 31; mean age 19.7 ± 3.2 years) consisting of individuals with overweight or obesity (BMI ≥ 25 kg/m^2^). There were no significant differences in age (*p* = 0.13) or sex distribution between groups.

Based on preliminary data, a sample size of 30 per group was calculated to detect a difference of 0.8 standard deviations in strain parameters with 80% power and *α* = 0.05 using two-tailed testing.

The study was conducted in accordance with the Declaration of Helsinki (2013 revision) and approved by the Ethics Committee of East Slovak Institute of CV Diseases (approval number: 2769341; date: 17 January 2023). All participants provided written informed consent after receiving detailed information about the study procedures, potential risks, and their right to withdraw at any time without consequences. For participants under 18 years of age (*n* = 8), additional parental/guardian consent was obtained.

### 2.2. Body Composition Assessment

Body composition was measured using multi-frequency bioelectrical impedance analysis (InBody 520; InBody Co., Seoul, Republic of Korea). This validated device uses eight-point tactile electrodes and measures impedance at six different frequencies (1, 5, 50, 250, 500, and 1000 kHz) to provide segmental body composition analysis. The InBody 520 has demonstrated excellent correlation with dual-energy X-ray absorptiometry (DXA) for body composition assessment (*r* = 0.95 for fat mass, *r* = 0.97 for lean mass) and test–retest reliability (ICC > 0.99).

Participants were instructed to fast for at least 4 h, avoid vigorous exercise for 12 h, empty their bladder immediately before measurement, and remove all metal accessories. Measurements were performed in the morning between 8:00 and 10:00 AM while participants stood barefoot on the device’s electrodes and held the hand electrodes with arms slightly abducted from the body. The device measured total body water, protein, minerals, body fat mass, and SMM. From these measurements, we calculated SMM percentage (SMM%) = [skeletal muscle mass (kg)/body weight (kg)] × 100, body fat %, lean body mass %, and mineral %.

In addition to standard body composition parameters, we calculated the SMM-to-Fat ratio as SMM-to-Fat ratio = SMM (%)/Body Fat (%). This ratio was used as a continuous variable throughout all analyses. The SMM-to-Fat ratio provides a single integrated metric that captures the balance between metabolically active lean tissue and adipose tissue [18,19]. Unlike percentage-based measures, which are inherently confounded by changes in body weight (the denominator effect), the ratio directly quantifies the proportional relationship between muscle and fat [17]. Higher ratios indicate greater muscle mass relative to fat mass, representing a more favorable body composition, while lower ratios indicate disproportionate adiposity relative to muscle mass.

The ratio approach has been increasingly utilized in body composition research due to several advantages: (1) it eliminates mathematical confounding inherent when both numerator and denominator share the same base (total body weight); (2) it provides improved statistical power by condensing correlated variables into a single metric; (3) it aligns with the biological concept that relative balance, rather than absolute quantities, drives metabolic outcomes, and (4) it demonstrates superior associations with cardiometabolic risk factors in multiple populations [18,19].

### 2.3. Biochemical Measurements

Fasting venous blood samples were collected in the morning (8:00–9:00 AM) after at least 8 h of overnight fasting. Samples were analyzed in the hospital’s certified clinical laboratory within 2 h of collection. We measured lipid profile (total cholesterol, triglycerides, HDL-cholesterol, LDL-cholesterol), liver enzymes (aspartate aminotransferase [AST], alanine aminotransferase [ALT], gamma-glutamyl transferase [GGT]), albumin, total protein, insulin, and C-peptide. All measurements were performed using standardized enzymatic methods on an automated analyzer (COBAS 6000, Roche Diagnostics, Basel, Switzerland). C-peptide and insulin were measured by electrochemiluminescence immunoassay with an intra-assay coefficient of variation < 5%.

### 2.4. Echocardiographic Assessment

Comprehensive transthoracic echocardiography was performed using a state-of-the-art ultrasound system (Philips EPIQ CVx, Philips Healthcare, Best, The Netherlands) equipped with an X5-1 matrix-array transducer. All examinations were conducted by experienced sonographers blinded to participants’ body composition data, following current guidelines [20].

Standard two-dimensional (2D) echocardiography included measurements of left ventricular dimensions, wall thickness, and left ventricular ejection fraction using the biplane Simpson’s method. Conventional parameters were recorded according to current recommendations.

For 2D speckle-tracking strain analysis, high-frame-rate images (60–80 fps) were acquired from apical four-chamber, two-chamber, and three-chamber views during breath-hold at end-expiration. The global longitudinal strain (GLS) was calculated as the average of peak systolic strain values from 18 segments across the three apical views [21].

For 3D strain analysis, full-volume 3D datasets were acquired from the apical window using ECG-gated multi-beat acquisition (4–6 cardiac cycles). Image quality was optimized to achieve clear endocardial and epicardial border definition with a frame rate > 20 volumes per second. The 3D datasets were analyzed offline using dedicated software—TomTec 4D LV-Analysis software version 3.1 (TOMTEC Imaging Systems, Unterschleissheim, Germany). Semi-automated endocardial border detection was performed with manual adjustments when necessary. The software (version 3.1) calculated 3D left ventricular longitudinal, circumferential, and radial strain based on tracking of speckle patterns throughout the cardiac cycle. All measurements were averaged from three consecutive cardiac cycles and performed by two independent observers, with excellent inter-observer agreement (ICC > 0.90).

Strain values are reported as negative values following echocardiographic convention. More-negative values indicate greater myocardial deformation (better function), while less-negative values (closer to zero) indicate impaired deformation (worse function). For example, a circumferential strain of −22% represents better cardiac function than −18%.

For quality control, we excluded datasets with poor image quality, insufficient temporal resolution, or tracking failure in >2 segments. All strain measurements were performed by a single investigator blinded to clinical and body composition data.

### 2.5. Statistical Analysis

Data are presented as mean ± standard deviation for continuous variables and numbers (percentages) for categorical variables. Normality of distribution was assessed using the Shapiro–Wilk test. Comparisons between groups were performed using independent samples *t*-test for normally distributed variables or Mann–Whitney U test for non-normally distributed variables. Categorical variables were compared using the chi-square test or the Fisher’s exact test.

We prospectively designated 3D global circumferential strain as the primary endpoint based on its established role in detecting mid-wall myocardial dysfunction in metabolic cardiomyopathy [22,23]. All other strain parameters (3D longitudinal strain, 3D radial strain, 2D global longitudinal strain) were designated as secondary exploratory endpoints. For the primary endpoint, statistical significance was set at *α* = 0.05 (two tailed).

Pearson correlation coefficients were calculated to examine associations between body composition parameters (as continuous variables) and cardiac strain measures. The SMM-to-Fat ratio was analyzed as a continuous variable throughout. For key associations, 95% confidence intervals for correlation coefficients were calculated using Fisher’s z-transformation.

Multivariable linear regression analysis was performed to identify independent predictors of 3D circumferential strain in the study group. To avoid overfitting, given our sample size (*n* = 31), we limited multivariable models to 3 predictors selected based on biological plausibility [9]: (1) body composition balance (SMM-to-Fat ratio as continuous variable), (2) metabolic function (C-peptide), and (3) age. This approach yielded an events-per-variable ratio of 10.3:1, meeting recommended thresholds [24].

Model assumptions were verified through examination of residual plots and assessment of influential observations. Multicollinearity was evaluated using variance inflation factors (VIFs), with VIF < 2 considered acceptable. All VIF values were below 1.1, indicating the absence of problematic multicollinearity.

All statistical analyses were performed using Python (version 3.8) with SciPy and scikit-learn libraries.

## 3. Results

### 3.1. Baseline Characteristics and Body Composition

Table 1 summarizes the baseline characteristics and body composition parameters. The two groups were well matched for age (19.7 ± 3.2 years vs. 21.0 ±2.9, *p* = 0.13) and sex distribution (*p* = 0.50). Anthropometric measurements differed significantly between groups. Weight (89.7 ± 16.3 kg vs. 61.9 ± 9.3 kg, *p* < 0.001) and BMI (30.6 ± 4.1 vs. 21.1 ± 2.2 kg/m^2^, *p* < 0.001) were substantially higher in the obese group.

Body composition analysis revealed profound differences. The control group exhibited significantly higher SMM % (38.0 ± 10.8% vs. 47.1 ± 5.6%, *p* < 0.001) while the study group had a markedly elevated body fat percentage (28.3 ± 12.6% vs. 16.0 ± 8.3%, *p* < 0.001).

Critically, the SMM-to-Fat ratio was substantially lower in the obese group (2.1 ± 2.3) than that in controls (4.8 ± 4.2, *p* < 0.001), reflecting a fundamentally unfavorable shift in body composition balance. The wide range of ratios within the obese group (0.53–11.27) indicates substantial heterogeneity in muscle–fat balance even among individuals with a similar BMI.

Other body composition parameters showed consistent patterns. Total body water percentage (51.5 ± 9.6% vs. 61.8 ± 6.3%, *p* < 0.001), protein percentage (14.1 ± 2.7% vs. 16.7 ± 1.8%, *p* < 0.001), and mineral percentage (3.5 ± 0.8% vs. 4.1 ± 0.6%, *p* = 0.001) were all significantly lower in the obese group.

### 3.2. Biochemical Parameters

C-peptide levels were significantly higher in the obese group (1242.6 ± 619.8 vs. 712.1 ± 385.8, *p* < 0.001), indicating compensatory hyperinsulinemia. Total cholesterol, triglycerides, and LDL cholesterol were elevated in the obese group, while HDL cholesterol was lower (all *p* < 0.01). Liver enzymes (AST, ALT, GGT) were within normal limits in both groups (Table 2).

### 3.3. Cardiac Function and Strain Analysis

#### 3.3.1. Primary Endpoint Analysis

The primary endpoint, 3D global circumferential strain, showed impairment in the obese group (less negative: −19.8 ± 4.7%) compared with controls (−22.2 ± 2.9%, *p* = 0.023), with a moderate-to-large effect size (Cohen’s d = 0.61) (Table 3). This represents an approximately 11% reduction in circumferential strain magnitude, indicating worse mid-myocardial function.

#### 3.3.2. Secondary Endpoints (Exploratory)

In contrast to circumferential strain, 2D global longitudinal strain showed no significant difference between groups (−22.8 ± 6.1% vs. −23.4 ± 4.2%, *p* = 0.64). Similarly, 3D LV longitudinal strain was preserved in both groups (−22.5 ± 6.1% vs. −23.1 ± 4.2%, *p* = 0.63). Moreover, 3D LV radial strain showed a trend toward reduction in the obese group (36.1 ± 5.2% vs. 38.6 ± 4.1%, *p* = 0.043), though this does not survive correction for multiple comparisons and should be interpreted cautiously. These findings indicate intact subendocardial function across both groups despite selective mid-wall impairment in obesity.

### 3.4. Body Composition Correlations: The Muscle-to-Fat Balance

#### 3.4.1. SMM-to-Fat Ratio: Superior Continuous Predictor

**Figure 1:**
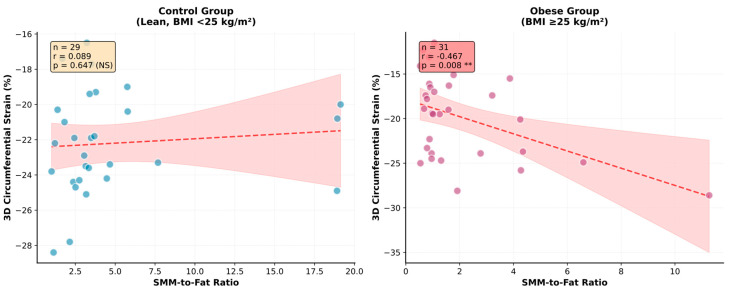
SMM-to-Fat ratio vs. circumferential strain by group. Control group on left side, study group on the right side. BMI = body mass index; 3D = three dimensional, SMM = skeletal muscle mass, NS = non significant, ** statistical significant.

**Figure 2:**
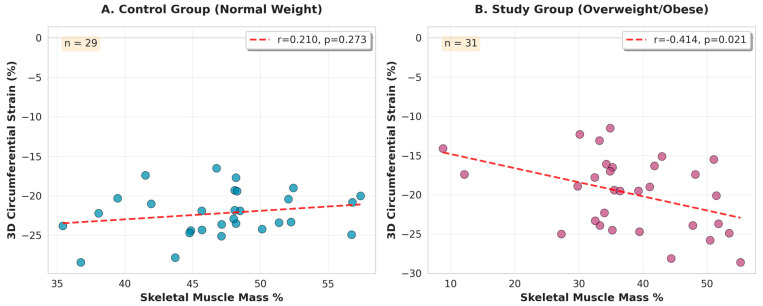
Skeletal muscle mass% vs. circumferential strain by group. Control group (**A**) on left side; study group (**B**) on the right side. BMI = body mass index; 3D = three dimensional, SMM = skeletal muscle mass.

The SMM-to-Fat ratio, analyzed as a continuous variable, demonstrated the strongest association with 3D circumferential strain in the obese group (*r* = −0.467, 95% CI: −0.704 to −0.134, *p* = 0.008), explaining 21.8% of variance in univariate regression (*R*^2^ = 0.218) (Figure 1). This correlation was significantly stronger than that observed for SMM% alone (*r* = −0.414, 95% CI: −0.670 to −0.070, *p* = 0.021, *R*^2^ = 0.171) or for body fat% (*r* = 0.264, *p* = 0.15), representing a 27% improvement in explained variance (Figure 2).

In the control group, body composition parameters showed weak and non-significant associations with circumferential strain (SMM%: *r* = 0.210, *p* = 0.27; SMM-to-Fat ratio: *r* = 0.089, *p* = 0.65), consistent with the expected minimal impact of normal-range body composition variation on cardiac function in healthy young adults.

#### 3.4.2. Correlation Reversal Across All Body Composition Parameters

Comprehensive correlation analysis revealed a striking pattern: all InBody body composition parameters showed reversal of correlation direction between groups. In the control group *(n* = 29), body composition parameters exhibited weak positive or null correlations with circumferential strain. However, in the obese group, these relationships completely reversed to negative correlations.

Key correlates in obese group (all continuous): 1. SMM-to-Fat ratio: *r* = −0.467, 95% CI: −0.704 to −0.134, *p* = 0.008; 2. minerals %: *r* = −0.452, 95% CI: −0.695 to −0.116, *p* = 0.011; 3. SMM %: *r* = −0.414, 95% CI: −0.670 to −0.070, *p* = 0.021; 4. lean body %: *r* = −0.376, 95% CI: −0.644 to −0.025, *p* = 0.037; 5. total body water %: *r* = −0.373, 95% CI: −0.642 to −0.021, *p* = 0.039.

To determine whether these associations were confounded by adiposity, we calculated partial correlations controlling for body fat % (Table 4). Minerals% (partial *r* = −0.42, *p* = 0.018), lean body% (partial *r* = −0.36, *p* = 0.046), and TBW% (partial *r* = −0.37, *p* = 0.040) remained significantly associated with circumferential strain after adjustment, indicating effects independent of fat mass. Importantly, the SMM-to-Fat ratio also maintained statistical significance (partial *r* = −0.42, *p* = 0.021) despite moderate attenuation, demonstrating superior robustness compared to SMM% alone. In contrast, the SMM% association was attenuated and lost statistical significance (partial *r* = −0.34, *p* = 0.065), suggesting that its effect is partially confounded by adiposity.

This comprehensive reversal pattern suggests fundamental differences in the body composition–cardiac biology relationship in obesity, extending beyond muscle mass alone to encompass multiple tissue compartments.

### 3.5. Multivariable Regression Analysis

In multivariable linear regression controlling for C-peptide and age (Table 5), the SMM-to-Fat ratio was a significant independent predictor of 3D circumferential strain (*β* = −0.876, SE = 0.350, 95% CI: −1.60 to −0.16, *p* = 0.019). For each 1-unit increase in the ratio, circumferential strain improved by 0.88% points. Neither C-peptide (*p* = 0.830) nor age (*p* = 0.143) reached significance. The model explained 20% of variance (adjusted *R*^2^ = 0.199, *F* (3.27) = 3.49, *p* = 0.029). All variance inflation factors were <2, indicating no multicollinearity.

When SMM% and body fat% were entered as separate predictors, neither achieved significance (SMM%: *β* = −0.136, 95% CI: −0.33 to 0.06, *p* = 0.159; body fat%: *β* = 0.024, 95% CI: −0.13 to 0.18, *p* = 0.757), with both confidence intervals including zero (Table 6). This model showed inferior performance: lower explanatory power (adjusted *R*^2^ = 0.093 vs. 0.199), lack of overall significance (*p* = 0.166), and a 6.4-fold smaller effect size for SMM%. The ratio approach demonstrated superior predictive validity across all criteria.

Two models were compared (Table 5 and Table 6): Model 1 using SMM-to-Fat ratio as a composite measure of body composition and Model 2 using SMM% and body fat% as separate predictors. Both models controlled for C-peptide and age. Only Model 1 achieved overall statistical significance and contained an individually significant predictor.

## 4. Discussion

### 4.1. Principal Findings and the Superiority of Muscle-to-Fat Balance

Our investigation in young obese adults yielded three key observations: (1) circumferential myocardial strain demonstrated selective dysfunction while longitudinal deformation remained intact; (2) the proportional relationship between skeletal muscle and adipose tissue (SMM-to-Fat ratio) provided superior cardiac risk stratification compared with muscle mass evaluated in isolation, accounting for 27% additional variance in outcome prediction; and (3) obesity fundamentally reorganized the associations between multiple body composition parameters and cardiac mechanics, affecting not only muscle and adipose compartments but also mineral content and total body water.

The enhanced predictive capacity of SMM-to-Fat ratio over isolated measurements aligns with established principles in body composition science. Studies examining relative muscle mass normalized to fat rather than absolute muscle mass demonstrate enhanced associations with cardiovascular outcomes [17,18]. Evidence indicates that the lean-to-fat mass ratio provides stronger prognostic information than either parameter assessed independently [19], corroborating our finding that the equilibrium between metabolically active and energy-storing tissues governs cardiac health beyond the simple quantification of individual components.

Multiple factors explain the ratio’s enhanced performance. Physiologically, the metric captures a fundamental biological reality: metabolic homeostasis reflects the equilibrium between insulin-responsive muscle tissue and inflammatory adipose depots rather than absolute tissue quantities [6]. Statistically, ratio construction optimizes the events-per-variable relationship, improving from 7.8:1 to 10.3:1, thereby satisfying the recommended criteria for robust multivariable regression modeling [24].

### 4.2. Selective Circumferential Strain Impairment: A Recognized but Less Common Pattern

Our documentation of preferential circumferential dysfunction with maintained longitudinal mechanics corresponds to a specific phenotype reported in advanced obesity states. Wierzbowska-Drabik and colleagues observed identical findings in severely obese individuals (mean BMI 46 ± 6): significant circumferential and radial deformation abnormalities coexisted with preserved longitudinal function [22]. This phenotypic expression appears particularly characteristic of younger populations with substantial adiposity [23], indicating heterogeneous manifestations of obesity-associated myocardial dysfunction.

This presentation contrasts with the predominant pattern documented in metabolic syndrome and moderate obesity, where longitudinal deformation typically demonstrates earliest impairment while circumferential mechanics remain relatively maintained [25,26]. The conventional pattern reflects preferential vulnerability of subendocardial fibers, where longitudinally oriented myocytes experience initial injury due to elevated metabolic requirements and heightened sensitivity to perfusion compromise [27]. The frequency of longitudinal-first impairment in the published literature suggests subendocardial dysfunction represents the initial myocardial response to metabolic perturbations in most cases.

The observation of two distinct phenotypes—longitudinal-predominant versus circumferential-predominant dysfunction—indicates that obesity-associated cardiac impairment represents a heterogeneous spectrum rather than a uniform progression. Several determinants may influence phenotypic expression: (1) Adiposity severity: advanced obesity (exemplified by Wierzbowska-Drabik’s cohort with BMI 46 ± 6) [22] may preferentially manifest circumferential dysfunction, whereas moderate obesity typically demonstrates longitudinal-first patterns. (2) Patient age and exposure duration: our youthful cohort (mean age 20 years) may exhibit distinct temporal progression compared with older populations with prolonged obesity exposure. (3) Hemodynamic alterations: circumferential deformation demonstrates particular sensitivity to ventricular configuration and loading pressures [21], which undergo substantial modification in severe obesity through expanded intravascular volume and elevated cardiac workload. (4) Layer-specific pathophysiology: contemporary imaging techniques reveal that the dominant pathophysiological mechanism—whether predominantly metabolic, inflammatory, or hemodynamic—may determine which myocardial layers experience earliest injury [28,29].

The biological mechanisms driving circumferential impairment in severe obesity likely involve multiple converging pathways. Lipotoxic injury from excessive fatty acid flux affects cardiomyocyte performance across myocardial layers [30], while chronic volume expansion and increased hemodynamic burden impose disproportionate mechanical stress on circumferentially oriented mid-wall fibers responsible for ejection mechanics. The metabolic disruptions characterizing young adults with severe obesity may therefore manifest distinctly from classical ischemic-pattern subendocardial injury observed in older populations with metabolic syndrome and multiple comorbidities.

### 4.3. Multi-Compartment Effects: Beyond Muscle and Fat

The comprehensive reorganization of body composition–cardiac function associations across all compartments—encompassing mineral content, total body water, protein fraction, and lean body mass—demonstrates that obesity fundamentally restructures the biological relationships between somatic composition and myocardial performance. Particularly noteworthy is the independent correlation between mineral percentage and circumferential strain (*r* = −0.45, *p* = 0.011; partial *r* = −0.42, *p* = 0.018 after adiposity adjustment), suggesting involvement of osseous–muscular–cardiac signaling axes whereby skeletal-derived factors influence both striated muscle tissues and cardiac function through endocrine communication [31,32].

### 4.4. Potential Mechanisms of Cardiac Dysfunction

Multiple interconnected mechanisms may underlie the paradoxical inverse association between elevated SMM-to-Fat ratio and impaired circumferential deformation in our obese population.

First, the pathophysiological disruption of adipokine–myokine equilibrium characteristic of sarcopenic obesity may facilitate myocardial lipid accumulation through augmented free fatty acid delivery and deposition of harmful lipid metabolites including ceramide species and diacylglycerol compounds within cardiac myocytes [18]. This lipid-overload environment can directly compromise mitochondrial bioenergetics and contractile protein function, with mid-myocardial layers demonstrating particular vulnerability due to elevated oxidative metabolic activity and heightened susceptibility to metabolic stress [33,34].

Second, the compensatory hyperinsulinemic state documented in our obese participants (75% higher C-peptide levels) may paradoxically promote myocardial insulin signaling resistance, disrupting physiological cardiac substrate metabolism [35]. Sustained insulin elevation impairs intracellular signaling cascades in cardiomyocytes, resulting in diminished glucose utilization and increased dependency on fatty acid catabolism, which demonstrates reduced bioenergetic efficiency and elevated reactive oxygen species generation [36].

Third, diminished muscle quantity and quality in sarcopenic obesity reduces secretion of beneficial myokines including irisin, interleukin-15, and insulin-like growth factor-1, which ordinarily provide cardioprotection through local and systemic signaling mechanisms [37,38]. Concurrently, expanded adipose tissue depots, particularly intra-abdominal fat, augment production of pro-inflammatory adipokines such as leptin, resistin, and tumor necrosis factor-alpha, establishing sustained low-grade systemic inflammation that directly injures myocardial tissue and compromises contractile performance [28,39].

### 4.5. Strengths and Limitations

The methodological strengths of this study include 3D speckle-tracking echocardiographic assessment providing comprehensive layer-specific cardiac evaluation; enrollment of young adults preceding extensive cardiovascular remodeling; validated bioelectrical impedance methodology [40]; continuous rather than categorical treatment of the SMM-to-Fat ratio throughout statistical analyses; prospective endpoint designation; and an adequate events-per-variable relationship (10.3:1) satisfying established statistical guidelines [24].

Several limitations warrant consideration. The cross-sectional design precludes determination of temporal relationships and causality. The sample size (*n* = 31 obese participants) constrains statistical power for subgroup evaluation, though it is adequate for primary analyses. Bioelectrical impedance demonstrates inherent limitations [40]; future investigations should incorporate reference methodologies including dual-energy X-ray absorptiometry or magnetic resonance imaging for body composition quantification and cardiac magnetic resonance for strain assessment. We did not measure aerobic fitness capacity or quantify circulating adipokine, myokine, or osteokine concentrations, which would provide mechanistic insights into observed associations. While our observed strain pattern corresponds to findings in severe obesity [22,23], it represents a less prevalent presentation than longitudinal-predominant patterns characteristic of metabolic syndrome [25,26], necessitating validation in independent populations to elucidate determinants of phenotypic expression.

### 4.6. Clinical Implications and Future Directions

Our observations carry important implications for cardiovascular risk stratification in young obese adults. The superior performance of proportional muscle–adipose metrics suggests clinical evaluation should prioritize relative measurements over absolute tissue quantification [17,18,19]. The accessibility and non-invasive nature of bioelectrical impedance technology enables practical comprehensive body composition screening in routine clinical settings [40]. Detection of subclinical myocardial dysfunction using advanced deformation imaging [28,29] supports incorporation of these modalities into screening protocols for high-risk populations. The osseous–muscular–cardiac axis associations [31,32] suggest comprehensive evaluation including skeletal health may enhance prognostic assessment.

Saltijeral and colleagues demonstrated in pediatric populations that circumferential strain exhibited the strongest obesity association in multivariable modeling (*β*-coefficient: 0.74; *r^2^*: 0.55; *p* = 0.003) [41], reinforcing our observation that this parameter may demonstrate the highest sensitivity for obesity-related cardiac dysfunction. Similarly, the Multi-Ethnic Study of Atherosclerosis documented strong associations between progressive left ventricular concentric remodeling and declining mid-wall circumferential deformation [42]. Recognition of distinct strain phenotypes in obesity—circumferential-predominant in severe cases [22,23] versus longitudinal-predominant in moderate metabolic dysfunction [25,26]—suggests phenotypic characterization may inform targeted therapeutic strategies.

Reversibility of obesity-associated cardiac dysfunction with weight reduction has been established [43]. Importantly, therapeutic approaches targeting both adipose reduction and muscle preservation may demonstrate superiority over isolated weight loss strategies, given our findings regarding proportional muscle–adipose balance importance. Future investigations should examine whether interventions specifically optimizing the SMM-to-Fat ratio produce greater cardiac functional improvements than weight-reduction-only approaches.

Additional research is needed to (1) identify clinical and pathophysiological determinants predicting individual patient strain phenotypes; (2) establish whether the SMM-to-Fat ratio independently predicts cardiovascular events in longitudinal cohorts; (3) elucidate mechanistic connections between mineral content, skeletal-derived factors, and cardiac dysfunction; (4) investigate roles of adipokines, myokines, and osteokines in mediating body composition effects on cardiac function; and (5) evaluate whether targeted interventions optimizing muscle-to-fat proportions can reverse subclinical cardiac dysfunction in young obese adults.

## 5. Conclusions

Young obese adults demonstrate selective circumferential strain dysfunction, a phenotype documented in severe obesity that differs from the more prevalent longitudinal-predominant impairment characteristic of metabolic syndrome. The SMM-to-Fat ratio provides superior cardiac dysfunction prediction compared with isolated muscle mass assessment, demonstrating a 27% enhanced explanatory capacity and independent predictive validity. Comprehensive body composition evaluation reveals multi-compartment effects extending beyond muscle and adipose tissues to encompass mineral content and total body water, suggesting complex mechanistic interactions involving osseous–muscular–cardiac signaling and fluid homeostasis. These findings support a conceptual shift from evaluating individual body composition components toward assessing the proportional equilibrium between metabolically active and energy-storing tissues. Early identification of unfavorable muscle-to-fat balance using accessible bioelectrical impedance methodology may enable targeted interventions preserving cardiac health before irreversible structural injury develops.

## Figures and Tables

**Table 1:** Baseline characteristics and body composition.

Variable	Study Group (*n* = 31)	Control Group (*n* = 29)	*p*-Value
Age (years)	19.7 ± 3.2	21.0 ± 2.9	0.1284
BMI (kg/m^2^)	30.6 ± 4.1	21.1 ± 2.2	<0.001
SMM (%)	38.0 ± 10.8	47.1 ± 5.6	<0.001
Body fat (%)	28.3 ± 12.6	16.0 ± 8.3	<0.001
SMM-to-Fat ratio	2.1 ± 2.3	4.8 ± 5.1	0.0087
Lean body (%)	71.1 ± 12.3	84.1 ± 8.4	<0.001
TBW (%)	51.5 ± 9.6	61.8 ± 6.3	<0.001
Minerals (%)	4.6 ± 0.9	5.6 ± 0.4	<0.001

Data presented as mean ± SD. *p* < 0.001 vs. control group. TBW = total body water; BMI = body mass index; SMM = skeletal muscle mass. Data presented as mean ± SD. *p* < 0.001 vs. control group.

**Table 2:** Biochemical parameters.

Parameter	Study Group (*n* = 31)	Control Group (*n* = 29)	*p*-Value
C-peptide (pmol/L)	1242.6 ± 619.8	712.1 ± 385.8	<0.001
Total cholesterol (mmol/L)	4.33 ± 0.64	4.25 ± 0.75	0.642
HDL-cholesterol (mmol/L)	1.26 ± 0.42	1.58 ± 0.42	0.001
LDL-cholesterol (mmol/L)	2.84 ± 0.72	2.55 ± 0.63	0.098
Triglycerides (mmol/L)	1.16 ± 0.55	0.79 ± 0.40	0.003

Data presented as mean ± SD. Exact *p*-values are shown in parentheses for significant differences vs. control group. HDL = high-density lipoprotein, LDL = low-density lipoprotein.

**Table 3:** Cardiac strain parameters.

Parameter	Study Group (*n* = 31)	Control Group (*n* = 29)	*p*-Value
2D GLS (%)	−22.8 ± 6.1	−23.4 ± 4.2	0.636
3D LV longitudinal strain (%)	−22.5 ± 6.1	−23.1 ± 4.2	0.627
3D LV circumferential strain (%)	−19.8 ± 4.7	−22.2 ± 2.9	0.023
3D LV radial strain (%)	36.1 ± 5.2	38.6 ± 4.1	0.043

Data presented as mean ± SD. GLS = global longitudinal strain; 3D = three dimensional, LV = left ventricule.

**Table 4:** Partial correlations controlling for body fat percentage.

Parameter	Original *r*	*p*-Value	Partial *r*	*p*-Value
Minerals%	−0.452	*p* = 0.011	−0.421	*p* = 0.020
SMM-to-Fat ratio	−0.467	*p* = 0.008	−0.421	*p* = 0.021
SMM%	−0.414	*p* = 0.021	−0.335	*p* = 0.070
Lean body%	−0.376	*p* = 0.037	−0.362	*p* = 0.050
TBW%	−0.373	*p* = 0.039	−0.371	*p* = 0.043

Partial correlations were performed controlling for body fat percentage to assess independent associations between body composition parameters and cardiac strain indices. All correlations are adjusted for body fat% as a covariate. SMM = skeletal muscle mass. TBW = total body water.

**Table 5:** Multivariable linear regression models predicting 3D circumferential strain in obese adolescents. Model 1: ratio approach.

Variable	*β*	95% CI	Standardized *β*	*p*-Value	VIF
SMM-to-Fat ratio	−0.876	(−1.595, −0.158)	−0.461	0.019	1.07
C-peptide (pmol/L)	0.0003	(−0.0023, 0.0029)	0.024	0.830	1.05
Age (years)	−0.364	(−0.859, 0.131)	−0.192	0.143	1.02

Model statistics: *n* = 31; *k* = 3 predictors; events per variable = 10.3:1; *R*^2^ = 0.279; adjusted *R*^2^ = 0.199; *F* (3.27) = 3.49; *p* = 0.029; *β*, unstandardized regression coefficient; CI, confidence interval; VIF, variance inflation factor.

**Table 6:** Multivariable linear regression models predicting 3D circumferential strain in obese adolescents. Model 2: separate components.

Variable	*β*	95% CI	Standardized *β*	*p*-Value	VIF
SMM%	−0.136	(−0.329, 0.057)	−0.321	0.159	1.54
Body fat%	0.024	(−0.134, 0.182)	0.055	0.757	1.42
C-peptide (pmol/L)	0.0002	(−0.0027, 0.0030)	0.013	0.904	1.13
Age (years)	−0.306	(−0.854, 0.242)	−0.161	0.262	1.10

Model statistics: *n* = 31; *k* = 4 predictors; events per variable = 7.8:1; *R*^2^ = 0.214, adjusted *R*^2^ = 0.093; *F* (4.26) = 1.77; *p* = 0.166 (NS); *β*, unstandardized regression coefficient; CI, confidence interval; VIF, variance inflation factor.

## Data Availability

The original contributions presented in this study are included in the article. Further inquiries can be directed to the corresponding author.

## Abbreviations

The following abbreviations are used in this manuscript:

2D	Two Dimensional
3D	Three Dimensional
BIA	Bioelectrical Impedance Analysis
BMI	Body Mass Index
BP	Blood Pressure
CI	Confidence Interval
CV	Cardiovascular
DXA	Dual-Energy X-Ray Absorptiometry
GCS	Global Circumferential Strain
GLS	Global Longitudinal Strain
HDL	High-Density Lipoprotein
LDL	Low-Density Lipoprotein
LV	Left Ventricular
SD	Standard Deviation
SMW	Skeletal Muscle Mass
TAG	Triglyceride
TBW	Total Body Water
VIF	Variance Inflation Factor
